# Video games as stimuli in neuroimaging studies: a minireview

**DOI:** 10.3389/fnhum.2026.1687121

**Published:** 2026-03-06

**Authors:** Isak B. Blank, Vasily Klucharev, Anna Shestakova

**Affiliations:** 1 International Laboratory of Social Neurobiology, National Research University Higher School of Economics, Moscow, Russia; 2 Center for Cognition and Decision Making, National Research University Higher School of Economics, Moscow, Russia

**Keywords:** cognition, emotions, functional magnetic resonance imaging, neuroimaging, perception, video games

## Abstract

In video games, the participants are active agents who pursue various goals within gaming environments that increasingly resemble real life. As a result, video games are increasingly offering tools for neuroimaging studies aiming to elucidate the neural basis of human perceptual, cognitive, and emotional functions. Here, we review these studies. The first studies used computerized versions of behavioral economic games during functional magnetic resonance imaging (fMRI) of brain activity, revealing brain structures relevant to mutual cooperation and structures responding when participants received unfair offers. Subsequently, first-person shooter games revealed brain activity differences during winning *vs*. losing. Video games have also proven useful for studying reward processing, cognitive processes during goal pursuit, and emotional responses within component models emphasizing active agency. Moreover, video games are especially well-suited for research on the neural basis of flow state. We also discuss shortcomings and ways forward in this exciting area of research.

## Introduction

Video games, while offering significant entertainment and educational value (e.g., via gamification of learning; Hong et al., 2024), also provide powerful tools for human neuroimaging studies. In contrast to movies used as naturalistic stimuli during neuroimaging—where participants are passive observers (Sonkusare et al., 2019; Vanderwal et al., 2019; Jaaskelainen et al., 2021)—video game participants actively engage in game play. This distinction is important because humans, like other organisms, are inherently goal-directed and active rather than passive perceivers (Basu and Nagel, 2024). Video games extend the possibilities to study the neural basis of human perceptual, cognitive, and emotional functions under complex and naturalistic stimuli and tasks approaching real-life conditions. At the same time, neuroimaging results might be relevant for game design.

The purpose of this minireview is to provide an overview of this exciting field of study from the perspective of how playing video games has been implemented in functional magnetic resonance imaging (fMRI) studies of perceptual, cognitive, and emotional functions. The majority of the previous review articles have focused on the long-term effects of video gaming on the brain, which we will not cover here. While some of them also covered studies in which video games were used as stimuli during fMRI (e.g., Palaus et al., 2017), recent review articles on the topic are lacking. As there is a range of complexity and naturalness of video games that have been used as stimuli in fMRI studies, we start by briefly introducing results from fairly simple games before describing the results obtained with more naturalistic video games.

## Economic games in the fMRI scanner

Economic games, such as the ultimatum, dictator, and prisoner’s dilemma games, have been developed and used in behavioral studies to study economic and social decision-making (Levitt and List, 2007; van Dijk and De Dreu, 2021). When adapted to the neuroimaging environment, they have helped gain an understanding of the cerebral basis of human cooperation and perception of fairness (see Wu and Hong, 2022).

In the ultimatum game, there are two players: one proposes how to divide a sum of money, and the other responds either by accepting or rejecting. Given this design, the ultimatum game has been relatively easy to adapt to the fMRI environment, and thus, not surprisingly, the first study was carried out more than two decades ago. In this study, responses in the anterior insula (AI) were observed when experimental participants received unfair offers in the ultimatum game (Sanfey et al., 2003). In a subsequent study, it was observed that unfair offers both to the players themselves and toward others whom they were playing on behalf of activated the AI, suggesting that AI supports perception of unfairness in general (Corradi-Dell’Acqua et al., 2013). In contrast, the ventromedial prefrontal cortex (VMPFC) only responded when the unfair treatment was directed against the self, and this effect was augmented for offers that the players rejected (Corradi-Dell’Acqua et al., 2013).

In a separate set of studies, participants played the Prisoner’s Dilemma game during fMRI. In this game, if both players choose to cooperate, they receive the same amount of money. If one player defects, the defector receives a larger sum than if both had cooperated. Additionally, the one who chose to cooperate in that round receives nothing. If both players defect, they get less than if both cooperate. During mutual cooperation, a significant increase in hemodynamic activity was observed in the striatal structures, the VMPFC, and the rostral anterior cingulate cortex (ACC) (Rilling et al., 2002). In contrast, when the other player defected while the participants chose to cooperate, there were robust responses in the AI (Rilling et al., 2008). In addition to the AI, activations were observed in the hippocampus and the amygdala, together with a deactivation of the ventral striatum (Rilling et al., 2008). Since defecting is considered unfair to the other player, the observed AI activity aligns with the results from the ultimatum game (Sanfey et al., 2003; Corradi-Dell’Acqua et al., 2013). This conclusion is further supported by findings from ultimatum game play in neurological patients with insula lesions (Nitsch et al., 2022).

## Cooperation vs. competition in a pattern-building game

In addition to classical economic games, brain mechanisms supporting competition vs. cooperation have been studied in a game in which players build target patterns with tokens (Decety et al., 2004). In the cooperation and competition conditions, it was possible both to help and block the other’s attempts in achieving the pattern, respectively. Brain hemodynamic activity was significantly stronger in the orbitofrontal cortex (OFC) during cooperation than during competition, which the authors interpreted as indicating that the cooperation condition was more rewarding (Decety et al., 2004). This is in line with the findings of VMPFC activity during cooperation in the prisoner’s dilemma game (Rilling et al., 2002). In the reverse contrast, brain areas showing enhanced activity during competition included the inferior parietal cortex and the superior medial prefrontal cortex, which were interpreted as signs of less self-other merging during the competition condition (Decety et al., 2004). Naturally, as reviewed elsewhere, cooperation and competition in the real world are impacted by a variety of factors, including cross-cultural differences and intergroup polarization (van Dijk and De Dreu, 2021).

## Board games

Board games are among the first video games used as stimuli in fMRI studies. In one study, players viewed in different conditions a blank board, a random board, and a game board of a so-called GO game. The participants, all of whom were experienced GO players, were instructed to consider the move they would make when viewing the game board. When brain activity during the game board viewing was contrasted with that during random board viewing, enhanced activity was observed in the dorsal prefrontal, parietal, posterior cingulate, occipital, and posterior temporal regions (Chen et al., 2003). Interestingly, these areas were highly similar to those observed during playing chess (Chen et al., 2003). While board games are not as naturalistic as some of the more recent video games, they nonetheless allow one to study strategic thinking (for a review on the neurobiology of strategic thinking, see Lee and Seo, 2016). Overall, simplified game designs could also help disentangle factors governing neurocognition that take place in more complex video game settings.

## First-person shooter games

First-person shooter games were the first video games used as stimuli with naturalistic graphics and flow of events in fMRI studies. They have provided a significant complementary tool for the study of aggression beyond the more conventional research methods, including the study of structural and functional resting-state connectivity changes in persons with trait aggression and experimental designs in which participants are passively viewing aggressive facial expressions (see Rosell and Siever, 2015).

In the first study, activation of the dorsal ACC, precuneus, cerebellum, and temporoparietal junction was observed during violent scenes, in addition to the suppression of activity in the rostral ACC, amygdala, intraparietal sulcus, OFC, posterior insula, and parahippocampal areas (Mathiak and Weber, 2006). The suppression of ACC and amygdala activity around the time of engaging in violence in the game was corroborated by a subsequent study (Weber et al., 2009a). It has been further suggested that the suppressive effects are predominantly seen in participants with a history of playing violent games, with naïve participants exhibiting an increase in activity during violent game play (Gentile et al., 2016).

In another study, medial prefrontal activity was observed during wins and losses during both passive observation and active game play; however, striatal responses were stronger during game play than during passive viewing of a recording of game play (Katsyri et al., 2013). This latter finding was corroborated by a subsequent study (Jaaskelainen et al., 2016). It was also observed that playing a first-person shooter game against a human opponent resulted in stronger responses in the striatum and the VMPFC than playing against a computer opponent (Katsyri et al., 2013). These findings somewhat contrast those of a previous study in which only the visual cortex responded to both wins and losses, with striatal structures only responding to losses (Mathiak et al., 2011).

In this area of research, methodological advances play a crucially important role, including the development of suitable experience sampling and content analysis methods to guide the neuroimaging data analyses (Klasen et al., 2008; Weber et al., 2009b). Using this type of approach, the ventral posterior cingulate cortex and theory of mind network of the brain were identified as central for the prediction of attack intents of others (Ye et al., 2025).

In an interesting recent study, experimental participants were immersed in a first-person shooter game while their supplementary motor area (SMA) activity levels were continuously monitored using fMRI (Baqapuri et al., 2020). The participants were told that self-inducing higher levels of SMA activity during game play would result in the avatar moving faster, thus yielding benefits in game play. This was successful, as the participants were taught to upregulate their SMA activity levels to aid their game play (Baqapuri et al., 2020). It is easy to envision how this type of setup might be translated to complement existing gamified rehabilitation paradigms in neurological patients (Ong et al., 2021).

## Video games and studies of reward structures

Cole et al. (2012) assigned participants to two groups: one group observed a recording, while the other played the interactive video game Re-Mission, designed for cancer patients to increase cancer awareness and promote adherence to chemotherapy. Compared with the observation condition, engagement in interactive game play increased activity in mesolimbic areas, including the caudate nucleus and the nucleus accumbens involved in reward processing (see Wang et al., 2016), as well as in the parahippocampal gyrus, thalamic, and insular areas. Perhaps a bit surprisingly, medial parietal and prefrontal activity decreased when the participants were engaged in game play vs. passive viewing.

These results show that active engagement robustly enhances activity in the mesolimbic reward areas compared with passive viewing of the same materials, which was interpreted as supporting previous behavioral findings of agency being a central factor that explains the efficacy of Re-Mission game play in the context of cancer treatment (Kato et al., 2008). These findings are also in line with observations of higher involvement of striatal structures in video game play *vs*. observation conditions (Katsyri et al., 2013; Jaaskelainen et al., 2016). Overall, these findings demonstrate how additional insights can be obtained via brain imaging on the mechanisms contributing to the efficacy of educational games such as Re-Mission.

## Video games in studies of the flow state

Another phenomenon that closely relates to reward processing is called the flow state (Csikszentmihalyi, 1975). Flow state can be defined as intense and pleasurable engagement in a task. While other tasks (e.g., arithmetic tasks) have also been used in neuroimaging studies of the flow state (see Alameda et al., 2022), captivating video games are ideal for inducing the flow state. This is because they enable the manipulation of psychological, cognitive, and behavioral variables implicated in the flow experience. Given its importance for motivated performance and the difficulties in capturing it with questionnaires, finding neural markers for flow state would also be of high application value (see Khoshnoud et al., 2020).

Flow state results when the task difficulty and individual ability are in balance: too difficult tasks tend to be anxiety-inducing, and too easy tasks are boring. When brain activity elicited during a balanced-difficulty task was contrasted with too difficult and too easy tasks, activity was observed in the dorsolateral prefrontal cortex (DLPFC), the superior parietal lobule, AI, and putamen, attributed to cognitive control, attention, and reward processing. Additionally, the DLPFC and putamen exhibited heightened functional connectivity during balanced task difficulty, suggesting that intrinsic reward and cognitive control are coupled during a flow state (Huskey et al., 2018). Interestingly, the DLPFC is also a hub in a brain network that supports the feeling of presence in virtual reality (Baumgartner et al., 2008; Jäncke et al., 2009). In contrast to balanced task difficulty, overly easy tasks resulted in activation of the default-mode network (DMN) (Huskey et al., 2018), consistent with findings suggesting that boredom during video gaming drives DMN activation (Mathiak et al., 2013).

In one study, the time courses of factors known to contribute to flow experience, i.e., balance between ability and challenges, concentration, direct feedback, clear goals, and control over the activity, were used as predictors of fMRI responses (Klasen et al., 2012). In this analysis, the balance between ability and challenge resulted in activity in the striatal structures, cerebellum, thalamus, superior parietal cortex, and motor–premotor areas. Concentration, in turn, was associated with increased activity in the cerebellum, visual areas, precuneus, and premotor cortex. During the presence of clear goals, activity was enhanced in the fusiform face area and the intraparietal sulcus. Finally, control was associated with the activity in the visual, cerebellar, thalamic, and motor regions (Klasen et al., 2012). Overall, these results revealed specific neural mechanisms supporting different constituents of the flow experience.

## Video games in studies of thoughts

Video games have been successfully used in neuroimaging of thoughts that took place while London cab drivers were navigating through virtual streets of London (Spiers and Maguire, 2006; for a review of the cerebral basis of human navigation, see Epstein et al., 2017). After game play, they were asked to self-report thoughts that they had in various phases of the game. This allowed the authors to investigate patterns of brain activity associated with customer-driven route planning, spontaneous route planning (e.g., to adapt to changing traffic), expectations, as well as their confirmation and violation, visual inspection, and traffic monitoring. When contrasted with coasting conditions, each elicited distinct brain activity patterns. During customer-initiated route planning, the hippocampus was recruited along with posterior parietal and dorso- and ventrolateral prefrontal cortices (Spiers and Maguire, 2006). These results illustrate the potential of video games combined with post-neuroimaging questionnaires in elucidating the neural basis of thought processes.

## Video games in studies of emotions

In the component process model of emotions (CPM), appraisal, motivation, physiology, and expression components dynamically interact to produce emotional states (Scherer, 2009). During video game play, the appraisal component, coping potential, and goal conduciveness recruited brain regions associated with action planning, uncertainty, valuation, reward processing, attention, and salience/relevance detection (Leitao et al., 2020). Motivation processes recruited the orbitofrontal, anterior cingulate, and posterior parietal cortices associated with approach and avoidance. The expression component, modeled with participants’ facial electromyography, and the physiology component, measured using heart rate and electrical skin conductance, co-varied with activity in areas associated with control of facial muscles as well as subcortical, prefrontal, and insular areas associated with affective interactions with peripheral physiology (Leitao et al., 2020).

The authors further identified somatomotor areas as being involved in synchronized changes across component processes during the emergence of emotional reactions (Leitao et al., 2020). This supports the notion that emotions are embodied action preparation mechanisms that help the organism to adapt to its environment. These results unveil the potential of video games to facilitate our understanding of human emotions in ways previously unexplored in neuroimaging studies, as previous studies have mostly been mapping reactions during the viewing of emotional stimuli (see Saarimaki, 2021). Notably, given that emotions are, in CPM, understood as arising in situations of active pursuit of goals among challenges, video games offer more suitable tasks/stimuli for neuroimaging studies of emotions in the context of CPM than traditional emotion elicitation paradigms in which the experimental participant passively perceives emotional stimuli.

## Video games in studies of social cognition

In an interesting study, players were engaged in either context-appropriate behaviors or context-inappropriate behaviors (King et al., 2006). Context-appropriate behaviors involved shooting an assailant and healing a wounded person. Context-inappropriate behaviors included shooting a wounded person and healing an assailant. During context-appropriate vs. context-inappropriate behaviors, enhanced activity was observed in the VMPFC and the amygdala, as well as in the AI (King et al., 2006). These findings were interpreted to support, in healthy subjects, the somatic marker hypothesis formulated based on neurological patients with VMPFC and amygdala lesions failing to behave in a contextually appropriate manner (see Damasio, 1994). These results in the healthy video game players suggest that areas supporting contextually appropriate behaviors additionally include the AI (King et al., 2006), though the AI activity could also indicate empathy to the pain of others (Singer et al., 2004).

## Limitations and future directions

Shortcomings in the literature include relatively small sample sizes in many studies that may have resulted in real effects not reaching statistical significance and prevented investigation of, for example, gender differences reported by some studies (Hoeft et al., 2008). Overall, in many areas of cognitive neuroscience, there have been relatively few studies that have used video games as stimuli, limiting the impact of the approach. Continued development of fMRI analysis methods, such as state space modeling approaches, is vital when using video games as stimuli in fMRI studies (Zhang et al., 2021). Furthermore, it has been shown that functional near-infrared spectroscopy (fNIRS) and fMRI provide similar results (Noah et al., 2015). The use of fNIRS would be beneficial since movement of participants is less restricted in fNIRS than in fMRI. This is important because spatial navigation relies on locomotion and proprioception that are lacking in the fMRI environment (Taube et al., 2013). As a shortcoming of fNIRS, imaging of deeper brain structures such as the hippocampus remains challenging, and one would have to rely on measuring activity of cortical areas that closely correlate with hippocampal activity (Jahani et al., 2017). Finally, video games are well suited for studying moral decisions that vary across contexts and cultures (Awad et al., 2018, 2020), therefore hold significant potential for neuroimaging investigations of moral decision-making.

## Conclusion

Video games have significantly extended the possibilities offered by other naturalistic stimulus paradigms, such as movies, by adding the dimension of the participants being active agents pursuing their goals. Video games used in neuroimaging studies have varied greatly, from simplified scenarios such as computerized versions of economic games to highly complex and naturalistic ones. Tailored video games can be used to study the neural basis of perceptual, cognitive, and emotional functions in novel and ecologically valid ways that would be otherwise difficult to achieve. Studies of the neural basis of the flow state and studies based on component models of emotions in which active agency is the key element offer good examples of research questions in which video games offer unique possibilities over more traditional experimental designs, given that the pursuit of these research questions requires active engagement from the participant.
